# Sensors and Technologies in Spain: State-of-the-Art

**DOI:** 10.3390/s140815282

**Published:** 2014-08-19

**Authors:** Gonzalo Pajares

**Affiliations:** Department of Software Engineering and Artificial Intelligence, Faculty of Informatics, University Complutense of Madrid, 28040 Madrid, Spain; E-Mail: pajares@ucm.es; Tel.: +34-1-394-7546; Fax: +34-1-394-7547

## 1. Introduction

The aim of this special issue was to provide a comprehensive view on the state-of-the-art sensor technology in Spain. Different problems cause the appearance and development of new sensor technologies and *vice versa*, the emergence of new sensors facilitates the solution of existing real problems.

The explosive technological advances and developments in recent years are evident. After the call for papers we received over a hundred of manuscripts, 88 of high quality, of which, were finally selected after a peer review process conducted by prestigious scientists with a high level of expertise in the different covered topics. A broad range of sensor-based technologies, procedures and applications are addressed, as a whole, which represent the state of the art in Spain.

The development, tests, and review make important contributions for the current information society, which demands the creation, distribution, and processing of information for different purposes, allowing some improvements from the point of view of economic aspects, including competitiveness and productivity, at the same time different issues of the human life are improved.

There are still many challenges and problems to be solved or improved, but the references provided in this special issue make an important progress in this regard, facilitating subsequent scientific and technological developments for industrial applications.

Some considerations about future trends are also provided, on the basis of the evolution of society and the new required services and demands.

The efforts of Spanish researchers is appreciated, who, together with other international teams, have facilitated the preparation of this special issue, which serves to the international scientific and industrial communities.

Three main areas are covered by this special issue, namely:

a) Specific sensors, *i.e.*, devices and technologies which are developed, exploiting some properties provided by materials or electrical and electronics elements;
b) Applications based on existing sensors, *i.e.*, systems or platforms that exploit the performances of sensors, which are integrated with a specific goal;
c) Methods, techniques, procedures, or algorithms specifically developed with some specific purpose of applicability, including simulation-based approaches.

Works providing specific reviews are also considered.

The proposals are not exclusively limited to sensors but also to different scientific fields, providing to the special issue a multidisciplinary nuance.

The classification of the published works according to these criteria is not easy, because many of them address more than one of the areas identified above. It, therefore, seems more appropriate to consider a categorization, attending to large areas of coverage given by the primary nature or main topic covered in the work. Thus, we have considered sixteen main topics, each one containing the most important subtopic.

However, it is still possible to separate the main topics into two categories depending on whether they are focused on the point of view of applications, based on sensors, or sensory-based technologies. The following is a list of the main topics according to each category.

**Applications Based on Sensors**

1. Networks: wireless sensors, web sensing, and semantic web.
2. Robots.
3. Sensors for analysis of surfaces and structures.
4. Temperature and humidity-based sensors.
5. Sensors in agriculture and biomass analysis.
6. Metrology.

**Sensory-Based Technologies**

7. Electric and electronic specific sensors.
8. Magnetic sensors.
9. Imaging-based systems: visible, multispectral, infrared, range, and ultrasonic.
10. Optical devices, lasers and lidars.
11. Radar devices.
12. Biosensors.
13. Internet and Internet of Things (IoT)
14. Field Programmable Gate Arrays (FPGAs).
15. Thermal sensors.
16. Multisensory technologies.

Tables 1 and 2 also contain the main topics expressed above, plus the sub-topics, with a brief description summarizing the content of each paper, for each one the associated reference is also provided in ascending order.

## 2. Applications Based on Sensors

### 2.1. Networks: Wireless Sensors, Web Sensing and Semantic Web

Vehicular *Ad Hoc* Networks (VANETs) are designed in [1] for cooperative driving among cars on the road. They allow communication between vehicles on the road with the aim of sharing information during traffic congestion, for route planning improvement, and safety, among others. This requires message dissemination among the dynamic nodes (cars). The goal is to determine the key factors affecting warning message dissemination to concentrate research tests on such factors.

Event-based wireless communication is proposed in [2], in an experimental environment with mobile robots, where each robot is considered as an agent. The mobile robots get their position through a camera, which performs as sensor. The video images are processed in a PC and a Waspmote card sends the corresponding position to each robot using the ZigBee standard. A distributed control algorithm, based on event-triggered communications, has been designed and implemented to bring the robots into the desired formation. Each robot communicates to its neighbors only at event times.

Extension of lifetime in Wireless Sensor Networks (WSN) is addressed in [3]. The key idea is that nodes equipped with renewable energy sources handle a higher number of messages than nodes with conventional batteries. A hierarchical network architecture is designed, similar to realistic scenarios, where nodes with renewable energy sources (denoted as primary nodes) carry out most message delivery tasks, and nodes equipped with conventional chemical batteries (denoted as secondary nodes) are those with less communication demands.

WSNs have been proven successfully in the Doñana Biological Reserve (Southern Spain) for monitoring this habitat. In [4], a review is presented of the work developed during the last five years, demonstrating the potential of using machine learning because of the amount of useful information supplied to biologists.

Operational and implementation details on low-power wireless communication technologies including time-slotted channel hopping (TSCH) and DASH7 Alliance Mode (D7AM) are provided in [5] with the aim of facilitating the adoption of these technologies to sensor application developers in industrial environments.

In [6], a WSN is designed with dynamic reconfiguration or redeployment for pre-emergency gas detection during ship-building, due to the high probability of existence of toxic gases. A wireless multihop remote gas monitoring system has been tested in a real ship under construction. Using this system, they validated the IEEE 802.15.4/Zigbee wireless networks as a suitable technology to connect gas detectors to control stations outside the ships.

The connectivity and reliability of WSNs is improved through a novel Fuzzy-logic topology control technique, where simulation results have verified that the k-Neighbor algorithm becomes effective [7].

Regarding sensor networks configuration, in [8], the problem of determining the optimal geometric configuration of sensor networks for multiple target positioning is tackled. The proposed approach is based on range measures coming from different sources based on the power of the signal. Optimality conditions for target positioning and for the sensor networks together with concepts and techniques from estimation theory and convex optimization are proposed for the best configurations.

In [9], several drift compensation mechanisms in WSN are analyzed. Temperature is a key factor affecting oscillators shipped in the WSN motes, producing low performances. Clock skew adjustments are required to compensate the adverse temperature effects. Mobile sensing systems are part of ubiquitous sensing, integrating other areas, such as WSN and web sensing.

In [10], a review is performed to provide a comprehensive overview of the current research on underwater WSNs, focusing on the lower layers of the communication stack, and envisions future trends and challenges. They analyze the current state-of-the-art of the physical, medium access control, and routing layers.

In the context of sensor networks, an advanced step is the integration of sensors into the web. Smart devices and connected objects acquire information about the environment and communicate it to other devices, objects, and people (Internet of Things, IoT). In [11], a review is provided about these topics, including types of mobile sensing (individual, participatory, opportunistic, crowd, social, *etc.*) and people or environment centered. In addition, the sensing domain (human, urban, vehicular, *etc.*) is revised, including social barriers (privacy, laws, *etc.*) that limit the social acceptance and technical aspects (energy, security, fusion, *etc.*).

In this regard, an architectural solution, based on a conceptual platform, is discussed in [12], which relies on Next Generation Networks to interconnect a Sensor Layer and the Sensor Web, and on Sensor Web Enablement (SWE) standards to provide interfaces between Sensor Web and IoT applications.

### 2.2. Robots

In [13], a novel method for the calibration of a parallel robot is presented, which allows accurate configurations. The main sensor is a camera, installed on the robot hand, which determines the relative position of the robot with respect to a spherical object fixed in the working area of the robot. A kinematic model of the robot is used to find a new group of parameters, which minimizes errors in the kinematic equations.

A fusion-based system for human detection, on board a mobile platform for autonomous surveillance, is proposed in [14], based on two sensors, a laser, and a visible camera. The laser provides range information, which, fused with the data supplied by the camera, conveniently classified by histogram analysis and support vector machines, serves for human detection. The system includes calibration techniques to optimally fuse the information.

In [15], a multi sensor fusion framework implementing an event based Kalman Filter to combine the measurements of a global sensor and an inertial measurement unit (IMU) on an event based schedule has been proposed. The event is defined to reflect the necessity of the global information, when the estimation error covariance exceeds a predefined limit. The experimental platforms are based on the LEGO Mindstorm NXT, and consist of a differential wheel mobile robot navigating indoors with a zenithal camera as global sensor, and an Ackermann steering mobile robot navigating outdoors with a GPS system.

Autonomous navigation is a key issue in robots. Robotized tractors in agriculture represent a challenge for autonomy in guidance tasks. In [16], a low-cost GPS receiver for positioning is used, which is a key issue in autonomy, where potential quantization errors are minimized by using the Kalman filter.

In [17], a complete spike-based architecture from a Dynamic Vision Sensor (retina) to a stereo head robotic platform is proposed. The architecture is divided into layers: retina, visual information processing, and trajectory generator, based on a neuroinspired algorithm (SVITE) that can be replicated into as many times as DoF the robot has. The aim is to reproduce intended movements performed by humans taking into account as many features as possible from the biological point of view. This paper fills the gap between current spike silicon sensors and robotic actuators by applying a spike processing strategy to the data flows in real time.

In [18], new algorithms for detecting and tracking mobile objects (DATMO) are presented by a multirobot system working collaboratively together This approach is intended for security and surveillance applications, where long distance range sensors are used in wide outdoor environments. Two movement detection algorithms have been developed for the detection of dynamic objects by using both static and/or mobile robots. The solution to the overall problem is based on the use of a Kalman filter to predict the next state of each tracked object.

In the context of mobile robots and based on vision sensors, in [19] are proposed and compared global appearance descriptors, with the aim of building maps for autonomous robots in indoor environments where the localization for navigation purposes becomes a key issue.

In [20], a variant approach to the monocular SLAM problem is presented in conjunction with a human-robot interaction (HRI) framework. This is achieved by modifying the delayed inverse-depth feature initialization SLAM (DI-D SLAM) taking advantage of data captured by a secondary camera handled by a human. The human explores an unknown environment with the robot and when their fields of view coincide, the cameras are considered a pseudo-calibrated stereo rig obtaining depth estimations, which are used for solving the DI-D monocular SLAM. The SURF features for matching are also studied.

In [21] a way to provide mobile robots with the ability-skill to detect objects is presented for semantic navigation. The use of current trends in robotics is analyzed with the possibilities to be exported to other platforms. Two methods to detect objects are proposed, contour detection and a descriptor based technique, and both of them are combined to overcome their respective limitations.

Upon the base of robot-sensor network cooperation techniques, where sensor nodes (beacons) are used as landmarks, in [22] a range-only (RO) scheme is presented, that actuates over the measurement gathering process using mechanisms that dynamically modify the rate and variety of measurements that are integrated in an extended Kalman filter SLAM with auxiliary particle filters.

In [23] a multi-sensor humanoid robotic head for human robot interaction is presented based on perception mechanisms and imitation of human expressions and emotions. The robot is equipped with a set of sensors and systems that intend to mimic human senses. The specific systems are: stereo vision, inertial sensor, stereo audio, RGB vision color camera and a depth sensor (infrared projector combined with a monochrome CMOS sensor).

In [24] a perception system based on audio and visual inspection is proposed for a humanoid robotic head. It is a bio-inspired approach that fuses the information provided by the sensors with which a mobile robot is equipped. The sensory information is fused with a Bayes inference for interactive robots, where the goal is to localize a person by processing visual and audio data.

### 2.3. Sensors for Analysis of Surfaces and Structures

An optical-based system is the sensor used in [25] with a non-contact interferometric technique for surface digitalization, known as conoscopic holography taking some advantages over optical techniques such as laser triangulation. The quality of a reconstructed surface is analyzed depending on its relative position within the working range under different combination of parameters.

In [26] a review of sensory techniques is provided for determining the quality of outgoing flat-rolled products in the steel industry based on the flatness. They include: (*a*) *contact sensors*, such as stressometers or piezoelectric devices; (*b*) *non-contact*, based on optical sensors (laser scanning, fringe projection and Moiré topography-based devices), electromagnetic or capacitive devices.

Surface roughness is critical in different technological systems, particularly in aeronautical materials, where modification of geometrical surface properties can cause instabilities. In this way, in [27] the use of a speckle method is proposed for measuring roughness on an unmanned aircraft wing. A laser beam under a given angle is directed onto the surface to be examined. The roughness is determined based on the angular speckle correlation method.

In [28], optical fibers with Bragg gratings are used to analyze impact and vibrations derived from impacts in aeronautical structures. An impact can produce microscopic fissures evolving towards fractures. Additionally, they use high-frequency interrogator to collect relevant information about the impacts.

Hull cleaning before repainting ships becomes a key operation in the maintenance of ships. In [29] the evolutionary development of sensor systems for the automation of the preparation process of ship hull surfaces is described, before the painting process is performed. Such evolution considers the development of new automatic technologies for coating removal, including elevation platforms, sensorized robotic systems or the XYZ Table proposed in the work.

In [30], a microprocessor-based sensor is developed for quality measurements of offset printing plates through different image analysis processes (binarization, cropping, edge and circle extraction). A camera is embedded on the board.

In [31], a method, based on phase-shift analysis of an ultrasonic signal, is applied to provide an integrated encoding system for authentication of the authorship of wooden panel paintings where a fingerprint is obtained. An ultrasonic pure tone is emitted by a transducer impinging on the rough surface of the painting. A receiver located at the same height as the emitter collects the acustic energy according to the roughness on the surface.

Plenoptic cameras use an array of micro-lenses providing different low image resolutions. In [32], a fast and specialized hardware implementation of a super-resolution algorithm is proposed to increase the resolution in plenoptic cameras to obtain 3D depth information and refusing from the scene upon the basis that the images are obtained with different perspectives. The algorithm has been designed for field programmable graphic array (FPGA) devices using VHDL.

### 2.4. Temperature and Humidity-Based Sensors

Data loggers are the devices used in Fernández-Navajas *et al.* [33] to monitor temperature and relative humidity based on multivariate thermo-hygrometric characterization of the archeological site of Plaza de l'Almoina (Valencia, Spain) for preventive conservation purposes.

In [34], the effect of temperature and relative humidity is analyzed, based on the changes produced by the heating system on the altarpiece in the *mudejar* church of Santa Maria (Ateca, Spain). A monitoring system of 15 temperature and 15 relative humidity sensors record the data. Analysis of variance (ANOVA) is the proposed statistical-based approach for this cultural heritage application.

In [35], novel temperature sensor architecture is proposed, based on the ratio of two leakage-dependent measures in two transistors. Such a ratio is highly dependent on the temperature, with important robustness against process variations.

### 2.5. Sensors in Agriculture and Biomass Analysis

In [36], the concept of gridded *crop biometrics* was introduced for agricultural analysis, based on observations confined to spaces with reduced dimensions and known positions to build prediction models. A set of traits are selected to construct predictive models for grape yield and quality, based on: penetrometers, for measuring soil resistance; GPS for points references and elevation data; a monochrome camera centered in the near infrared band for vegetation quantification; a digital dynamometer for weighting the grapes; a digital refractometer to determine the sugar content of the must; a digital caliper for measuring the diameter of the grapes; acidity and pH are measured with a semi-automatic tritator and ph-meter respectively.

A review in the field of sensors, for characterizing vineyard canopies and monitoring spray drift, is provided in [37] in order to improve vineyard spraying. Different sensors are mentioned, including ultrasonic sensors, digital cameras, stereoscopic systems, or LIDAR scanners. Some methods and geostatistical procedures for mapping vineyard parameters are proposed, and the development of a variable rate sprayer is described. All these technologies are of interest in terms of adjusting the amount of pesticides applied to the target canopy.

Sensory and actuation systems are integrated in autonomous agricultural vehicles for site-specific tasks in Emmi *et al.* [38]. Such autonomous agricultural systems are integrated as whole perception systems for acquiring information from the environment, decision-making systems for interpreting and analyzing such information, and actuation systems that are responsible for performing the agricultural operations.

A spectroradiometer for analysis of aboveground biomass in grasslands is used in [39]. The spectral range covers 350 to 2,500 nm, where a set of data is collected for analysis based on support vector machines and partial least squares regression.

### 2.6. Metrology

The mechanical design of an indexed metrology platform (IMP) is proposed in [40]. The aim of the IMP is to increase the final accuracy and to radically simplify the calibration, identification and verification of geometrical parameter procedures of Portable Coordinate Measuring Machines (PCMMs) in industry, gaining flexibility for accomplishing in-line measuring tasks as compared to traditional coordinate measuring machines (CMMs).

The work in [41] proposed a laboratory prototype for ultra-precision metrology based on a new transducer with electronic interfacing. It is capable of sensing displacements in the micrometer range based on radio frequency resonant cavities, where the output frequency is captured by a low pass filter and two RF power detectors.

### 2.7. Electric and Electronic Specific Sensors

Energy consumption constraints on computing systems represent a key issue to balance performance *vs.* consumption for energy efficient applications. In [42], the most remarkable alternatives to measure the power consumption of different types of computing systems are provided. Measuring external and internal devices are proposed for such purpose. The first ones based on an intelligent programmable power supply (IPS) and the second one embedded in the computing system to be tested.

An electronic tongue, based on an array of potentiometric electrodes (gold, silver, copper and chemical compounds), is designed in [43] to classify different kinds of honeys. This device is connected to a microcontroller where data patterns are conveniently processed based on a simplified fuzzy ARTMAP neural network. It is also suggested that this kind of procedures can be successfully used, not only for honey classification, but also for many other kinds of food.

In [44], the design and implementation of a new real-time frequency sensor used as a hardware countermeasure has been presented. It is implemented in an Application-Specific Integrated Circuit (ASIC) to prevent attacks related to the clock frequency. These attacks are produced in the communication channels between emitter and receptor, or because of the weaknesses in algorithms with the aim of achieving important information about the system.

In medicine patient monitoring is a key issue. In [45] a smart sensor to monitor the respiratory rate has been designed and developed. It is based on capacitive sensing trough an LC oscillator.

In [46] the performance of two inductive sensors with different frequency responses to pulsed signals has been analyzed, a high frequency current transformer and an inductive loop sensor for detecting and separating sources of partial discharges as a standardized technique to qualify electrical insulation in machines and power cables.

In [47] the electrical characteristics of solution-processed organic photodetectors based on poly(3-hexylthiophene-2,5-diyl) semiconducting polymer layers deposited by spin-coating on interdigitated metal electrodes, with different geometries, has been described. The resistance as a function of the temperature reveals a transition from negative to positive temperature coefficient material for the polymer layers. Slow reversible changes in the photodetectors conductivity are observed when moved from vacuum to the air and under illumination with a xenon lamp, explained by the formation of charge transfer complexes with molecular oxygen and the polymer.

In [48] a sensor for quantitative assessment of the corneal barrier function for a correct corneal homeostasis has been proposed, where the corneal epithelial permeability is assessed *in vivo* by means of non-invasive tetrapolar impedance measurements. This is achieved by using a flexible sensor based in the impedance SU-8 photoresist polymer. This device was fabricated on a silicon wafer with high technology and connected to a printed circuit board (PCB). The electrode-electrolyte impedance has been assessed on physiological saline solution.

Based on flexible printing electronics, in [49] a pressure sensor sheet consisting of a 16 × 16 array with spatial resolution of 2 cm^2^ of the tactile unit and a total area of 45 cm^2^ × 45 cm^2^ has been designed. Each single sensor is formed by silver interdigitated electrodes defined by screen-printing technology on a polyethylene terephthalate flexible sustrate an poly (2,3-dioxythiophene) conducting polymer on the top side of the electrodes acting as an active coating. An insulating acrylate elastomeric coating is used as a separator between the electrodes and the electroactive coating. Data acquisition embedded software and hardware was developed using a Rapid Control Prototyping. The electronic prototype was improved by developing a Taylor-made electronics.

### 2.8. Magnetic Sensors

Based on the Hall effect, in [50] an array sensory device where a floating magnet in a milk volume meter allows detecting the magnetic field variation as the volume varies has been designed. This device has been used for monitoring milk yield during milking in goats.

Microelectromechanical (MEMS) Magnetic, Angular Rate and Gravity (MARG) sensors have increased in use because many commercial devices use them (smartphones, tablets, GPS navigators, watches, or videogame peripherals among others). Calibration of such systems is critical; with such a purpose, in [51], two ellipsoid-fitting calibration algorithms are proposed. The first approach is based on a thresholding algorithm that uses only one indicator as its input and the second one is based on a Fuzzy Logic System (FLS).

In [52], the experimental AC large-signal frequency response of a family of electrical current sensors based in different spintronic conduction mechanisms is shown. The transimpedance sensor function is obtained considering it as the relationship between sensor output voltage and input sensing current. The study has been extended to various magnetoresistance sensors, based on different technologies like anisotropic magnetoresistance (AMR), giant magnetoresistance (GMR), spin-valve (GMR-SV), and tunnel magnetoresistance (TMR).

In [53], a new displacement-to-frequency transducer based on the variation of a coil inductance has been designed, when a magnetic core is partially or completely inserted inside. The transducer is based on a Colpitts oscillator, which is robust against noise. The sensor has a dynamic range equal to the length of the coil. The cores can exchange sensors (coils with its ferromagnetic core) using the same electronic measuring system. In this way a unique electronic circuit suffices. The sensor is useful to measure structural vibrations in buildings.

In [54] the outstanding properties of selected soft magnetic materials with their electric or elastic properties that can be applied to build two kind of sensing technologies are exploited. The first is based on the influence on the magneto-impedance response of the thickness of Permalloy films in multilayer-sandwiched structures. The second technology is based on the magneto-elastic resonance of amorphous ribbons to demonstrate the possibility of sensitively measuring the viscosity of fluids with the aim to develop a real-time sensor capable of assessing the state of degradation of lubricant oils in machinery.

### 2.9. Imaging-Based Systems: Visible, Multispectral, Infrared, Range and Ultrasonic

In [55] a fusion strategy is proposed, it is based on color and depth information for background subtraction in video sequences, with the aim of detecting elements in the scene other than the background. Camera and active depth-based sensors are used for such purpose.

RGB and multispectral imagery are combined in [56] for discrimination of cabernet sauvignon grapevine elements (leaves, stems, branches, fruits. and background). A CCD-based camera and a servo-controlled filter wheel for the acquisition of images and a sequential masking algorithm based on the K-means was the design of this system.

In [57], a, RGB-D imaging sensor is used for human-computer interaction based on hand gestures. This technology provides information about the RGB color space for the elements in the scene, at the same time they provide depth information. Based on those data and semantic information the algorithm identified any number of hands in the scene with their associated gestures. In the field of phased array (PA) ultrasonic imaging systems one of the challenges is their limited capability to deal with real-time applications, such as echocardiography and obstetrics. These systems require emitting and receiving with the entire array for each image line to be acquired, limiting the acquisition time and frame rate.

In [58], a survey is provided on model-based approaches for 2D and 3D visual human pose recovery in the scope of computer vision with single and multi-imagery sensors. They also established a taxonomy in this regard.

In [59], a comparative analysis is performed, based on different methods used for the registration of RGB and depth video frames in static scenarios. The analysis begins by explaining the characteristics of the registration problem, dividing it into two representative applications: scene modeling and object reconstruction.

An infrared camera is used in [60] to adjust the required parameters, so that the flame front reaches the bottom of the bed, known as the burn-through point (BTP) during the industrial sintering process that applies heat to fine particles of iron ore to obtain a coarse grained product. The application is based on the extraction of relevant features based on a previous image segmentation process.

In [61], the design of an advanced driver assistance system (ADAS) is proposed for driver drowsiness detection. A NIR illuminator and a camera, sensitive to the NIR, are placed in front of the driver, in the dashboard, in order to acquire images of the driver's face independently of the ambient lighting conditions. When the lighting is low, the NIR illuminator automatically turns on and *vice versa*. Image processing algorithms are used for eye detection and tracking and eye closure parametrization to estimate some driver drowsiness clues. Driving data values from the vehicle, such as lateral position, heading error, steering wheel are combined with the above through an ANN. The system is tested in simulation as the step prior to real testing.

In [62], the Kinect depth sensor is used to obtain a high-quality foreground segmentation almost independently of the existing illumination conditions for indoor environments. They applied background subtraction, based on a Bayesian network, for such a purpose with surveillance applications.

In [63], a device for photogrammetry has been designed, equipped with a set of integrated sensors, including an accurate real-time kinematics global navigation satellite system (RTK-GNSS) used at construction sites, a prism for interfacing with a robotic total station, a high resolution camera and an inertial measurement unit (IMU). It moves between two mounting points, in a blondin ropeway configuration, at the construction site, taking pictures and recording the position and orientation data along the cable. The measurements provided by these sensors are integrated through a Kalman filter. Digital surface models (DSMs), based on the structure from motion (SfM), have been created at a construction site.

Based on the Code Division Multiple Access (CDMA) technique, in [64], an approach to solve the problem of the limited capability to deal with real-time applications (echocardiography and obstetrics) in phased array ultrasonic imaging systems is proposed. It is carried out by assigning a different code to each steering direction, allowing the array to emit in several directions simultaneously. The use of encoding techniques produces a reduction of the image contrast because of the interferences between codes. To solve this, a new scheme based on merging several images is proposed, allowing the system to get close to the theoretical maximum frame rate, as well as to limit the loss of contrast intrinsic to the technique.

### 2.10. Optical Devices, Lasers and Lidars

In [65], the design and development of a low-cost optical current sensor is presented, including its electronic. It uses plastic components (plastic optical fiber, collimators, and polarizers), where low current values can be measured with minimal errors. Potential uses are also described, including the control of electrolysis for production of metals, current monitoring on overhead electrical distribution lines, underground electrical vaults, currents within Switchgear, monitoring magnetic fields, and fault detection, among others.

The concept of sensor sharpening is addressed in [66], which was developed to achieve computational color constancy using a diagonal model in contrast with the human color constancy. They provide a review of methods used to obtain spectrally sharpened sensors under different categories. Additionally, they describe different research lines where sharpened sensors have not been successfully applied including chromatic adaptation, perceptual-based definition of color spaces, or hue equilibrium among others.

A 3D laser and CMOS-vision scanner is the device proposed in [67] for determining 3D measurements. Based on the well-known active triangulation principle, the laser projects a pattern on the surface to be measured, this pattern is captured on the focal plane of the camera, where applying triangulation the depth of the surface is obtained. Both, laser and camera are installed on a pant-tilt unit.

A terrestrial light detection and ranging (LIDAR) sensor is used in [68] for accuracy evaluation during maize and weeds discrimination. The system uses distance and reflection measurements for such purpose. Statistical-based analyses were the methods used for assessment in order to determine the correlation between the real growth state of plants and the measurements.

### 2.11. Radar Devices

In [69], a methodology is presented for automatic target recognition, based on Inverse Synthetic Aperture Radar (ISAR) with the generation of high-resolution imagery.

LFM (Linear Frequency Modulated) radars signals, coming from the corresponding transceiver and arranged as a radar network, are analyzed onboard dumpers in [70]. They proposed the use of Offset Linear Frequency Modulated Continuous Wave (OLFM-CW) as a radar signal. The goal is to improve safety conditions in the working environment.

### 2.12. Biosensors

The efficient potentiometric, sensitive and semiconducting properties, provided by InN/InGaN quantum dots, are exploited in [71] to design a low-dimensional biosensor for enzymatic bio-molecules sensing, such as cholesterol oxidase (ChOx), which is the most commonly employed enzyme in cholesterol biosensors because of its high selectivity to cholesterol molecules.

In [72], a surface plasmon resonance sensor has been designed, based on optical transmission through sub-wavelength nanohole arrays. Monitorization of protein absorption onto gold surfaces in a real-time label-free has demonstrated the biosensing capabilities of the device.

In [73], a review is provided on recent progress in Circulating Tumor Cell (CTC) identification using different approaches based on sensor signaling. CTCs are cells released from the primary tumor into the bloodstream, which are considered the main promoters of metastasis. A range of biosensor platforms are considered into the technology for CTC quantification.

### 2.13. Internet and Information Communication Technologies (ICT)

A framework for integrating utilities infrastructures and a Future Internet (FI) smart city is proposed in [74]. They exploit the concepts from the FI paradigm addressing the challenges to be overtaken when smart cities are created. Information and Communication Technologies play an important role. The design is based on the following five principles: (i) integration of heterogeneous infrastructure; (ii) Internet of things (IoT)-like large-scale network; (iii) dynamic network management; (iv) Sensing as a Service; and (v) cloud of services.

In the field of IoT combined with lifelogging sensors, the work in [75] proposed to build a Digital Memory (Dig-Mem) to complement the memory of people. The design is dimensioned based on sensors incorporated in smartphones that can be used to capture information. The following basic dimensions are selected: *thing* (DOI measured using RFID or NFC tag readers), *time* (measured with the reader or the smartphone clock), and *location* (using GPS positioning services).

### 2.14. Field Programmable Gate Arrays (FPGAs)

In [76], a methodology for designing an artificial neural network, as an equalizer for a binary signal, is proposed. After its modeling in Matlab, the design is based on the specifications of an FPGA using fixed point format. The FPGA design is based on the System Generator from Xilinx, which is a design tool over Simulink of Matlab.

The work in [77] proposed a novel self-timed multi-purpose delay sensor for FPGAs, which, through the use of asynchronous logic, carries out a delay measurement without the need of an external clock. The sensor generates a pulse of which the width is the amplification of the delay of a signal going through a delay-chain.

The work in [78] described the implementation of a web server using an embedded Altera NIOS II IP core, with a configurable RISC processor which is embedded in a Cyclone FPGA. The processor uses the μCLinux operating system to support a Boa web server of dynamic pages using Common Gateway Interface (CGI). The FPGA is configured to act as the master node of a network, and also to control and monitor a network of smart sensors or instruments. In order to develop a totally functional system, the FPGA also includes an implementation of the time-triggered protocol (TTP/A).

A review of developments for a new generation of smarter, reconfigurable and lower power consumption sensors, based on FPGAs, is provided in [79]. The FPGA technologies used for such purposes are also included.

In [80], a time derivative algorithm for estimation of signals is implemented in an FPGA, improving the speed of the system and achieving real-time performance. The derivative plays an important role in signal processing and control engineering. A non-asymptotic algebraic procedure for the approximate estimation is the proposed approach.

### 2.15. Thermal Sensors

A thermal sensor has been added to the Kinect device in [81] with the aim of detecting people from the view-scope of robots. The thermal information is added to the visual and depth information provided by the Kinect. A hierarchical approach combining supervised classifiers is the proposed strategy.

The work in [82] proposed a real-time temperature control system based on infrared (IR) thermometry for domestic induction cooking. The temperature sensor is based in the detection of the infrared radiation by an extended InGaAs PIN photodiode with spectral responsivity up to 2.600 nm.

In [83], a review of infrared thermography is provided and especially focused on two applications: temperature measurement and non-destructive testing, two of the main fields where infrared thermography-based sensors are used. A general introduction to infrared thermography and the common procedures for temperature measurement and non-destructive testing are presented. The intensity of the infrared radiation emitted by objects is mainly a function of their temperature. In infrared thermography, this feature is used for multiple purposes: as a health indicator in medical applications, as a sign of malfunction in mechanical and electrical maintenance, or as an indicator of heat loss in buildings.

### 2.16. Multisensory Technologies

In [84], the interest is focused on driver assistant systems intended to prevent undesired events, such as pedestrian detection. Multisensory technology is integrated onboard two autonomous vehicles for such a purpose. The sensors are an accurate high definition GPS with inertial measurement for localization and two sensors used for pedestrian detection (laser scanner and computer vision). Additionally, both vehicles are equipped with gateways communication devices that provided communication channel vehicles. Data provided by laser scanners are used for shape estimation with classification at a first level of detection. The vision system uses this information to confirm the presence of pedestrians, based on histogram of oriented gradients and support vector machines. The tracking is carried by a Kalman filter.

The work in [85] has addressed the slow sensor problem in control-based applications, where the sensor technology used to the measurement of the variable to be controlled is not able to maintain a restricted sampled period. In multirate (MR) systems special care must considered in this regard. MR systems are hybrid composed of continuous elements, some discrete time components or filters where the discrete actions are not equally spaced on time and/or delayed. An interactive simulation tool to deal with MR is proposed.

In [86], an interactive virtual laboratory is described for experimenting with an outdoor tubular photobioreactor (PBR). It is inspired by a real plant for microalgae cultivation for different applications, such as energy production, food, or medicine among others. In the industrial PBR is endowed with different measurement and performance units based on several sensors grouped as follows: temperature, flow, pH, dissolved oxygen, and turbidity of the concentration of biomass.

The work in [87] proposed a multisensory-based system to assess in real time the emotional, physical, and mental stress load of a combatant during combat. It is based on the use of wearable non-invasive physiological measurement provided by a set of sensors: galvanic skin response, body temperature, electrocardiogram, thoracic electrical bioimpedance, and voice. These sensors are designed to be integrated in a vest at a second development phase including signal processing algorithms with wireless communication.

The work in [88] addressed the minimum time search problem in uncertain domains in searching tasks, which appear in real world problems, such as natural disasters and sea rescue operations, where a target has to be found, as soon as possible, by a set of sensor-equipped searchers. On the basis that time is critical, different simulation tests have been carried out, based on several sensors models (radar, sonar, and cameras) in static and dynamic scenarios that can be easily extended to multisensory systems. The proposed solution consisted on exploiting the detection capabilities of the sensors to implement a Minimum Time Search (MTS) strategy that minimizes the Expected Time (ET) to detect targets using Cross Entropy Optimization.

## Figures and Tables

**Table 1.** Applications based on sensors: main topics and subtopics covered by the special issue.

**APPLICATIONS BASED ON SENSORS**			

	**Main Topic**	**Sub-Topics**	**Ref.**
1	*Networks: Wireless Sensors, Web Sensing and Semantic Web*	Vehicular *Ad Hoc* networks	[1]
		Event-based wireless communication with mobile robots	[2]
		Hierarchical networks based on different energy sources	[3]
		Habitat monitoring with wireless sensor networks	[4]
		Low-power wireless communications	[5]
		WSN dynamic reconfiguration for gas detection during ship building	[6]
		WSN connectivity and reliability	[7]
		Geometric configuration of sensor networks for multi-target positioning	[8]
		Drift compensation mechanisms in WSN	[9]
		Overview of the current research on underwater WSNs	[10]
		Mobile sensing systems (simulated review)	[11]
		Architectures for sensors in semantic web and Internet of Things	[12]
2	*Robots*	Parallel robot calibration with a camera on the robot hand	[13]
		Human detection by laser and camera in a mobile platform	[14]
		Sensor fusion in mobile robots navigating indoors and outdoors	[15]
		Autonomous tractor guidance in agriculture	[16]
		Architecture for dynamic vision sensor (stereo) and actuators in robots	[17]
		Detecting and tracking objects in a multirobot system	[18]
		Mapping through global descriptors based on vision for mobile robots	[19]
		Monocular SLAM and human robot interaction for depth estimations	[20]
		Object detection methods in mobile robots	[21]
		Range-only scheme on robot-sensor network cooperation techniques	[22]
		Multi-sensor humanoid robotic head for human robot interaction	[23]
		Bio-inspired video and audio perception for a humanoid robotic head	[24]
3	*Sensors for Analysis of Surfaces and Structures*	Conoscopic holography for surface digitalization	[25]
		Review of sensory techniques for quality of flat-rolled products	[26]
		Laser beam for roughness measurement on unmanned aircraft wings	[27]
		Optical fibers for impact analysis in aeronautical structures	[28]
		Hull cleaning for surface ships repainting	[29]
		Offset printing plates quality with a microprocessor-based sensor	[30]
		Authorship of wooden panel paintings based ultrasonic phase-shift	[31]
		Super-resolution in plenoptic cameras for 3D depth determination	[32]
4	*Temperature and Humidity-Based Sensors*	Data loggers for thermo-hygrometric analysis in archeology	[33]
		Temperature and relative humidity analysis in a church altarpiece	[34]
		Temperature sensor architecture based in two transistors	[35]
5	*Sensors in Agriculture and Biomass Analysis*	Crop biometrics for mapping based on several sensors	[36]
		Overview of sensors in vineyard canopies and monitorization	[37]
		Sensory and actuation integration in autonomous agricultural vehicles	[38]
		Spectroradiometer for analysis of biomass in grasslands	[39]
6	*Metrology*	Mechanical design of an indexed metrology platform	[40]
		Laboratory prototype for ultra-precision metrology	[41]

**Table 2.** Sensory-based technologies: main topics and subtopics covered by the special issue.

**SENSORY-BASED TECHNOLOGIES**			

	**Main Topic**	**Sub-Topics**	**Ref.**
7	*Electric and Electronic Specific Sensors*	Power consumption measurements in computing systems	[42]
		Electronic tongue for honey classification	[43]
		Real-time frequency sensor used as hardware countermeasure	[44]
		Capacitive sensing trough an LC oscillator to monitor patient respiration	[45]
		Inductive sensors for determining electrical insulation	[46]
		Electrical characteristics of solution-processed organic photodetectors	[47]
		Non-invasive tetrapolar impedance corneal barrier measurements	[48]
		Tactile pressure sensor	[49]
8	*Magnetic Sensors*	Hall effect for milk variation determination	[50]
		Calibration of MEMS Magnetic, Angular Rate and Gravity systems	[51]
		Large-signal frequency response in magnetoresistance sensors	[52]
		Displacement-to-frequency transducer based on Colpitts oscillator	[53]
		Magneto-impedance response and magneto-elastic resonance	[54]
9	*Imaging-BASED systems: Visible, Multispectral, Infrared, Range and Ultrasonic*	Fusion-based on color and depth information in video sequences	[55]
		Multispectral imagery for discrimination elements in grapevine	[56]
		RGB and depth range for human-computer interaction with hand gestures	[57]
		Model-based approaches survey for 2D and 3D visual human pose	[58]
		Comparison registration methods for RGB and depth video frames	[59]
		Infrared imaging in industrial sintering process	[60]
		NIR illuminator with camera for driver assistance	[61]
		Kinect depth sensor for high-quality foreground segmentation	[62]
		Photogrammetry with a high resolution camera and other sensors	[63]
		Phased array ultrasonic imaging systems for real-time applications	[64]
10	*Optical Devices, Lasers and Lidars*	Optical sensors for current measurements	[65]
		Review of sensor sharpening for computational color constancy	[66]
		3D laser and CMOS-vision scanner for 3D measurements	[67]
		LIDAR for maize and weeds discrimination	[68]
11	*Radar Devices*	Inverse Synthetic Aperture Radar for target recognition	[69]
		Linear Frequency Modulated radar signals for security	[70]
12	*Biosensors*	Properties for enzymatic bio-molecules sensing	[71]
		Surface plasmon resonance for monitorizing protein absorption	[72]
		Review of progress in Circulating Tumour Cells (CTCs) identification	[73]
13	*Internet and IoT*	Framework for integrating utilities and a Future Internet smart city	[74]
		Digital Memory to complement the memory of people	[75]
14	*Field Programmable Gate Arrays (FPGAs)*	Methodology for designing an artificial neural network	[76]
		Self-timed multi-purpose delay sensor	[77]
		Implementation of a web server	[78]
		Review of new generation of sensors based on FPGAs	[79]
		Derivative algorithm for estimation of signals in an FPGA	[80]
15	*Thermal Sensors*	Thermal sensor has been added to the Kinect	[81]
		Real-time temperature control system, based on infrared	[82]
		Review of infrared thermography	[83]
16	*Multisensory Technologies*	Driver assistant systems intended to prevent undesired events	[84]
		Slow sensor problem in control-based applications	[85]
		Virtual laboratory for experimenting in a photobioreactor	[86]
		Assessing the emotional, physical, and mental stress in a combatant	[87]
		Minimum time search problem in uncertain domains	[88]

## References

[1] Fogue M., Garrido P., Martinez F.J., Cano J.-C., Calafate C.T., Manzoni P. (2013). Identifying the key factors affecting warning message dissemination in VANET real urban scenarios. Sensors.

[2] Guinaldo M., Fábregas E., Farias G., Dormido-Canto S., Chaos D., Sánchez J., Dormido S. (2013). A mobile robots experimental environment with event-based wireless communication. Sensors.

[3] Asorey-Cacheda R., García-Sánchez A.J., García-Sánchez F., García-Haro J., González-Castano F.J. (2013). On maximizing the lifetime of wireless sensor networks by optimally assigning energy supplies. Sensors.

[4] Larios D.F., Barbancho J., Sevillano J.L., Rodríguez G., Molina F.J., Gasull V.G., Mora-Merchan J.M., León C. (2013). Five years of designing wireless sensor networks in the doñana biological reserve (Spain): An applications approach. Sensors.

[5] Vilajosana X., Tuset-Peiro P., Vazquez-Gallego F., Alonso-Zarate J., Alonso L. (2014). Standardized low-power wireless communication technologies for distributed sensing applications. Sensors.

[6] Pérez-Garrido C., González-Castaño F.J., Chaves-Díeguez D., Rodríguez-Hernández P.S. (2014). Wireless remote monitoring of toxic gases in shipbuilding. Sensors.

[7] Huang Y., Martínez J.-F., Díaz V.H., Sendra J. (2014). A novel topology control approach to maintain the node degree in dynamic wireless sensor networks. Sensors.

[8] Moreno-Salinas D., Pascoal A.M., Aranda J. (2013). Optimal sensor placement for multiple target positioning with range-only measurements in two-dimensional scenarios. Sensors.

[9] Castillo-Secilla J.M., Palomares J.M., Olivares J. (2013). Temperature-Compensated clock skew adjustment. Sensors.

[10] Climent S., Sanchez A., Capella J.V., Meratnia N., Serrano J.J. (2014). Underwater acoustic wireless sensor networks: Advances and future trends in physical, MAC and routing layers. Sensors.

[11] Macias E., Suarez A., Lloret J. (2013). Mobile sensing systems. Sensors.

[12] Díaz Pardo de Vera D., Sigüenza Izquierdo Á., Bernat Vercher J., Hernández Gómez L.A. (2014). A Ubiquitous sensor network platform for integrating smart devices into the semantic sensor web. Sensors.

[13] Traslosheros A., Sebastián J.M., Torrijos J., Carelli R., Castillo E. (2013). An inexpensive method for kinematic calibration of a parallel robot by using one hand-held camera as main sensor. Sensors.

[14] Fotiadis E.P., Garzón M., Barrientos A. (2013). Human detection from a mobile robot using fusion of laser and vision information. Sensors.

[15] Marín L., Vallés M., Soriano Á., Valera Á., Albertos P. (2013). Multi sensor fusion framework for indoor-outdoor localization of limited resource mobile robots. Sensors.

[16] Gomez-Gil J., Ruiz-Gonzalez R., Alonso-Garcia S., Gomez-Gil F.J. (2013). A kalman filter implementation for precision improvement in low-cost GPS positioning of tractors. Sensors.

[17] Perez-Peña F., Morgado-Estevez A., Linares-Barranco A., Jimenez-Fernandez A., Gomez-Rodriguez F., Jimenez-Moreno G., Lopez-Coronado J. (2013). Neuro-Inspired Spike-based motion: From dynamic vision sensor to robot motor open-loop control through Spike-VITE. Sensors.

[18] Rodríguez-Canosa G., del Cerro Giner J., Barrientos A. (2014). Detection and tracking of dynamic objects by using a multirobot system: application to critical infrastructures surveillance. Sensors.

[19] Payá L., Amorós F., Fernández L., Reinoso O. (2014). Performance of global-appearance descriptors in map building and localization using omnidirectional vision. Sensors.

[20] Guerra E., Munguia R., Grau A. (2014). Monocular SLAM for autonomous robots with enhanced features initialization. Sensors.

[21] Astua C., Barber R., Crespo J., Jardon A. (2014). Object detection techniques applied on mobile robot semantic navigation. Sensors.

[22] Torres-González A., Martinez-de Dios J.R., Ollero A. (2014). An adaptive scheme for robot localization and mapping with dynamically configurable inter-beacon range measurements. Sensors.

[23] Cid F., Moreno J., Bustos P., Núñez P. (2014). Muecas: A multi-sensor robotic head for affective human robot interaction and imitation. Sensors.

[24] Viciana-Abad R., Marfil R., Perez-Lorenzo J.M., Bandera J.P., Romero-Garces A., Reche-Lopez P. (2014). Audio-Visual perception system for a humanoid robotic head. Sensors.

[25] Fernández P., Blanco D., Rico C., Valiño G., Mateos S. (2014). Influence of surface position along the working range of conoscopic holography sensors on dimensional verification of AISI 316 wire EDM machined surfaces. Sensors.

[26] Molleda J., Usamentiaga R., García D.F. (2013). On-Line flatness measurement in the steelmaking industry. Sensors.

[27] Salazar F., Barrientos A. (2013). Surface roughness measurement on a wing aircraft by speckle correlation. Sensors.

[28] Gomez J., Jorge I., Durana G., Arrue J., Zubia J., Aranguren G., Montero A., López I. (2013). Proof of concept of impact detection in composites using fiber bragg grating arrays. Sensors.

[29] Navarro P.J., Muro J.S., Alcover P.M., Fernández-Isla C. (2013). Sensors systems for the automation of operations in the ship repair industry. Sensors.

[30] Poljak J., Botella G., García C., Poljaček S.M., Matías M.P., Tirado F. (2013). Offset printing plate quality sensor on a low-cost processor. Sensors.

[31] Bravo J.M., Sánchez-Pérez J.V., Ferri M., Redondo J., Picó R. (2014). Application of ultrasound phase-shift analysis to authenticate wooden panel paintings. Sensors.

[32] Pérez J., Magdaleno E., Pérez F., Rodríguez M., Hernández D., Corrales J. (2014). Super-Resolution in plenoptic cameras using FPGAs. Sensors.

[33] Fernández-Navajas Á., Merello P., Beltrán P., García-Diego F.-J. (2013). Multivariate thermo-hygrometric characterisation of the archaeological site of plaza de l'Almoina (Valencia, Spain) for preventive conservation. Sensors.

[34] García-Diego F.-J., Fernández-Navajas Á., Beltrán P., Merello P. (2013). Study of the effect of the strategy of heating on the mudejar church of santa maria in ateca (Spain) for preventive conservation of the altarpiece surroundings. Sensors.

[35] Ituero P., López-Vallejo M., López-Barrio C. (2013). A 0.0016 mm2 0.64 nJ leakage-based CMOS temperature sensor. Sensors.

[36] Rovira-Más F., Sáiz-Rubio V. (2013). Crop biometric maps: The key to prediction. Sensors.

[37] Gil E., Arnó J., Llorens J., Sanz R., Llop J., Rosell-Polo J.R., Gallart M., Escolà A. (2014). advanced technologies for the improvement of spray application techniques in spanish viticulture: An overview. Sensors.

[38] Emmi L., Gonzalez-de-Soto M., Pajares G., Gonzalez-de-Santos P. (2014). Integrating sensory/actuation systems in agricultural vehicles. Sensors.

[39] Marabel M., Alvarez-Taboada F. (2013). Spectroscopic determination of aboveground biomass in grasslands using spectral transformations, support vector machine and partial least squares regression. Sensors.

[40] Avila A.B., Mazo J.S., Martín J.J.A. (2014). Design and mechanical evaluation of a capacitive sensor-based indexed platform for verification of portable coordinate measuring instruments. Sensors.

[41] Asua E., Etxebarria V., García-Arribas A., Feutchwanger J., Portilla J., Lucas J. (2014). A novel micro- and nano-scale positioning sensor based on radio frequency resonant cavities. Sensors.

[42] Calandrini G., Gardel A., Bravo I., Revenga P., Lázaro J.L., Toledo-Moreo F.J. (2013). Power measurement methods for energy efficient applications. Sensors.

[43] Garcia-Breijo E., Garrigues J., Sanchez L.G., Laguarda-Miro N. (2013). An embedded simplified fuzzy ARTMAP implemented on a microcontroller for food classification. Sensors.

[44] Jiménez-Naharro R., Gómez-Galán J.A., Sánchez-Raya M., Gómez-Bravo F., Pedro-Carrasco M. (2013). Design and implementation of a new real-time frequency sensor used as hardware countermeasure. Sensors.

[45] Luis J.A., Roa Romero L.M., Gómez-Galán J.A., Hernández D.N., Estudillo-Valderrama M.Á., Barbarov-Rostán G., Rubia-Marcos C. (2014). Design and implementation of a smart sensor for respiratory rate monitoring. Sensors.

[46] Ardila-Rey J.A., Rojas-Moreno M.V., Martínez-Tarifa J.M., Robles G. (2014). Inductive sensor performance in partial discharges and noise separation by means of spectral power ratios. Sensors.

[47] Ferrer J.C., Alonso J.L., de Ávila S.F. (2014). Electrical characterization of photodetectors based on Poly(3-hexylthiophene-2,5-diyl) layers. Sensors.

[48] Guimerà A., Illa X., Traver E., Herrero C., Maldonado M.J., Villa R. (2014). New trends in quantitative assessment of the corneal barrier function. Sensors.

[49] Flores-Caballero A., Copaci D., Blanco M.D., Moreno L., Herrán J., Fernández I., Ochoteco E., Cabañero G., Grande H. (2014). Innovative pressure sensor platform and its integration with an end-user application. Sensors.

[50] García-Diego F.-J., Sánchez-Quinche A., Merello P., Beltrán P., Peris C. (2013). Array of hall effect sensors for linear positioning of a magnet independently of its strength variation. A case study: Monitoring milk yield during milking in goats. Sensors.

[51] Olivares A., Ruiz-Garcia G., Olivares G., Górriz J.M., Ramirez J. (2013). Automatic determination of validity of input data used in ellipsoid fitting MARG calibration algorithms. Sensors.

[52] Ravelo Arias S.I., Ramírez Muñoz D., Sánchez Moreno J., Cardoso S., Ferreira R., Peixeiro de Freitas P.J. (2013). Fractional modeling of the AC large-signal frequency response in magnetoresistive current sensors. Sensors.

[53] García A., Morón C., Tremps E. (2014). Magnetic Sensor for building structural vibrations. Sensors.

[54] García-Arribas A., Gutiérrez J., Kurlyandskaya G.V., Barandiarán J.M., Svalov A., Fernández E., Lasheras A., de Cos D., Bravo-Imaz I. (2014). Sensor applications of soft magnetic materials based on magneto-impedance, magneto-elastic resonance and magneto-electricity. Sensors.

[55] Fernandez-Sanchez E.J., Diaz J., Ros E. (2013). Background subtraction based on color and depth using active sensors. Sensors.

[56] Fernández R., Montes H., Salinas C., Sarria J., Armada M. (2013). Combination of RGB and multispectral imagery for discrimination of cabernet sauvignon grapevine elements. Sensors.

[57] Palacios J.M., Sagüés C., Montijano E., Llorente S. (2013). Human-Computer interaction based on hand gestures using RGB-D sensors. Sensors.

[58] Perez-Sala X., Escalera S., Angulo C., González J. (2014). A survey on model based approaches for 2D and 3D visual human pose recovery. Sensors.

[59] Morell-Gimenez V., Saval-Calvo M., Azorin-Lopez J., Garcia-Rodriguez J., Cazorla M., Orts-Escolano S., Fuster-Guillo A. (2014). A comparative study of registration methods for RGB-D video of static scenes. Sensors.

[60] Usamentiaga R., Molleda J., Garcia D.F., Bulnes F.G. (2013). Monitoring sintering burn-through point using infrared thermography. Sensors.

[61] Daza I.G., Bergasa L.M., Bronte S., Yebes J.J., Almazán J., Arroyo R. (2014). Fusion of optimized indicators from advanced driver assistance systems (ADAS) for driver drowsiness detection. Sensors.

[62] del-Blanco C.R., Mantecón T., Camplani M., Jaureguizar F., Salgado L., García N. (2014). Foreground segmentation in depth imagery using depth and spatial dynamic models for video surveillance applications. Sensors.

[63] Cuesta F., Lopez-Rodriguez F.M., Esteban A. (2013). A new blondin system for surveying and photogrammetry. Sensors.

[64] Gutiérrez-Fernández C., Jiménez A., Martín-Arguedas C.J., Ureña J., Hernández Á. (2014). A novel encoded excitation scheme in a phased array for the improving data acquisition rate. Sensors.

[65] Zubia J., Casado L., Aldabaldetreku G., Montero A., Zubia E., Durana G. (2013). Design and Development of a low-cost optical current sensor. Sensors.

[66] Vazquez-Corral J., Bertalmío M. (2014). Spectral sharpening of color sensors: Diagonal color constancy and beyond. Sensors.

[67] Vegara F., Zuccarello P., Boluda J.A., Pardo F. (2013). Taking advantage of selective change driven processing for 3D scanning. Sensors.

[68] Andújar D., Rueda-Ayala V., Moreno H., Rosell-Polo J.R., Escolá A., Valero C., Gerhards R., Fernández-Quintanilla C., Dorado J., Griepentrog H.-W. (2013). Discriminating crop, weeds and soil surface with a terrestrial LIDAR sensor. Sensors.

[69] López-Rodríguez P., Fernández-Recio R., Bravo I., Gardel A., Lázaro J.L., Rufo E. (2013). Computational burden resulting from image recognition of high resolution radar sensors. Sensors.

[70] González-Partida J.-T., León-Infante F., Blázquez-García R., Burgos-García M. (2014). On the use of low-cost radar networks for collision warning systems aboard dumpers. Sensors.

[71] Ul Hassan Alvi N., Gómez V.J., Soto Rodriguez P.E., Kumar P., Zaman S., Willander M., Nötzel R. (2013). An InN/InGaN quantum dot electrochemical biosensor for clinical diagnosis. Sensors.

[72] Martinez-Perdiguero J., Retolaza A., Otaduy D., Juarros A., Merino S. (2013). Real-Time label-free surface plasmon resonance biosensing with gold nanohole arrays fabricated by nanoimprint lithography. Sensors.

[73] Costa C., Abal M., López-López R., Muinelo-Romay L. (2014). Biosensors for the detection of circulating tumour cells. Sensors.

[74] Sánchez L., Elicegui I., Cuesta J., Muñoz L., Lanza J. (2013). Integration of utilities infrastructures in a future internet enabled smart city framework. Sensors.

[75] Arcega L., Font J., Cetina C. (2013). Towards memory-aware services and browsing through lifelogging sensing. Sensors.

[76] Suárez S.T.P., González C.M.T., Hernández J.B.A. (2013). Design methodology of an equalizer for unipolar non return to zero binary signals in the presence of additive white gaussian noise using a time delay neural network on a field programmable gate array. Sensors.

[77] Osuna C.G., Ituero P., López-Vallejo M. (2014). A self-timed multipurpose delay sensor for field programmable gate arrays (FPGAs). Sensors.

[78] Magdaleno E., Rodríguez M., Pérez F., Hernández D., García E. (2014). A FPGA embedded web server for remote monitoring and control of smart sensors networks. Sensors.

[79] García G.J., Jara C.A., Pomares J., Alabdo A., Poggi L.M., Torres F. (2014). A survey on FPGA-Based sensor systems: towards intelligent and reconfigurable low-power sensors for computer vision, control and signal processing. Sensors.

[80] Morales R., Rincón F., Gazzano J.D., López J.C. (2014). Real-Time algebraic derivative estimations using a novel low-cost architecture based on reconfigurable logic. Sensors.

[81] Susperregi L., Sierra B., Castrillón M., Lorenzo J., Martínez-Otzeta J.M., Lazkano E. (2013). On the use of a low-cost thermal sensor to improve kinect people detection in a mobile robot. Sensors.

[82] Lasobras J., Alonso R., Carretero C., Carretero E., Imaz E. (2014). Infrared sensor-based temperature control for domestic induction cooktops. Sensors.

[83] Usamentiaga R., Venegas P., Guerediaga J., Vega L., Molleda J., Bulnes F.G. (2014). Infrared thermography for temperature measurement and non-destructive testing. Sensors.

[84] García F., Jiménez F., Anaya J.J., Armingol J.M., Naranjo J.E., de la Escalera A. (2013). Distributed Pedestrian detection alerts based on data fusion with accurate localization. Sensors.

[85] Salt J., Cuenca A., Palau F., Dormido S. (2014). A multirate control strategy to the slow sensors problem: An interactive simulation tool for controller assisted design. Sensors.

[86] Dormido R., Sánchez J., Duro N., Dormido-Canto S., Guinaldo M., Dormido S. (2014). An interactive tool for outdoor computer controlled cultivation of microalgae in a tubular photobioreactor system. Sensors.

[87] Seoane F., Mohino-Herranz I., Ferreira J., Alvarez L., Buendia R., Ayllón D., Llerena C., Gil-Pita R. (2014). Wearable biomedical measurement systems for assessment of mental stress of combatants in real time. Sensors.

[88] Lanillos P., Besada-Portas E., Lopez-Orozco J.A., de la Cruz J.M. (2014). Minimum time search in uncertain dynamic domains with complex sensorial platforms. Sensors.

